# *Trichophyton indotineae* in Virginia: diagnostic identification and case report

**DOI:** 10.1128/asmcr.00058-25

**Published:** 2026-02-27

**Authors:** Hope Winfield, Nicole Edmonds, Wilson Omesiete, Richard Flowers, Amy Mathers, Emily Snavely

**Affiliations:** 1 University of Virginia School of Medicine 12349, Charlottesville, Virginia, USA; 2 Department of Dermatology, University of Virginia School of Medicine 12349, Charlottesville, Virginia, USA; 3 Department of Medicine, University of Virginia School of Medicine 12349, Charlottesville, Virginia, USA; 4 Department of Pathology, University of Virginia School of Medicine 12349, Charlottesville, Virginia, USA; Pattern Bioscience, Austin, Texas, USA

**Keywords:** ITS sequencing, itraconazole, *Trichophyton mentagrophytes*, antifungal, tinea corporis, terbinafine resistance

## Abstract

**Background:**

*Trichophyton indotineae* is an emerging *Trichophyton* species associated with extensive dermatophytosis frequently demonstrating terbinafine resistance. While early cases were reported in South Asia, *T. indotineae* has since spread globally, with several cases reported in the United States since 2023. To our knowledge, we describe the first cases reported in Virginia.

**Case Summary:**

Patients A and B each presented to the dermatology clinic with a diffuse, pruritic rash unresponsive to previous courses of topical and antifungal therapy. Both presentations raised concern for *T. indotineae* infection, which was confirmed with internal transcribed spacer (ITS) genome sequencing of isolates from the lesions. Both patients were initially prescribed an 8-week course of itraconazole 200 mg daily. Patient A required an additional month of treatment, but both patients had improved at follow-up.

**Conclusion:**

The present cases highlight several key points regarding the epidemiology, diagnosis, and management of *T. indotineae* infections, including continued expansion within the US, the need for ITS sequencing to distinguish *T. indotineae* from *Trichophyton mentagrophytes* and *Trichophyton interdigitale*, and the growing role of oral itraconazole as first-line therapy.

## INTRODUCTION

Dermatophytosis is a common superficial fungal infection targeting the skin, hair, and nails most often caused by species of the *Trichophyton* genus (1–4). These infections are commonly treated with topical or oral antifungal agents (1). However, a growing public health concern has emerged over the past decade due to the rapid global spread of *Trichophyton indotineae*, a newly recognized, antifungal-resistant species within the *T. mentagrophytes/interdigitale* complex (1–6).

First described in South Asia and now documented worldwide, *T. indotineae* poses diagnostic and therapeutic challenges (5–9). It is difficult to distinguish from other dermatophytes by microscopy, culture, or MALDI-TOF mass spectrometry (MS) and requires sequencing of the internal transcribed spacer (ITS) region for definitive identification. It is also frequently resistant to terbinafine, a common first-line oral antifungal, and treatment failures are increasingly reported (5, 10).

In recent years, clinical cases of *T. indotineae* have emerged globally, including United States (US) cases in New York and Pennsylvania gaining recognition in 2023 (2) and 2024 (1), respectively. To our knowledge, we report the first cases of *T. indotineae* in Virginia.

## CASE PRESENTATION

A 29-year-old immunocompetent male (Patient A) presented to the dermatology clinic in October of 2024 with a diffuse, itchy rash. He had immigrated to the US from Honduras in 2020, developing a rash seven months later. Emergency department visits resulted in trials of topical steroids, topical antifungals, and oral fluconazole without resolution.

**Fig 1:**
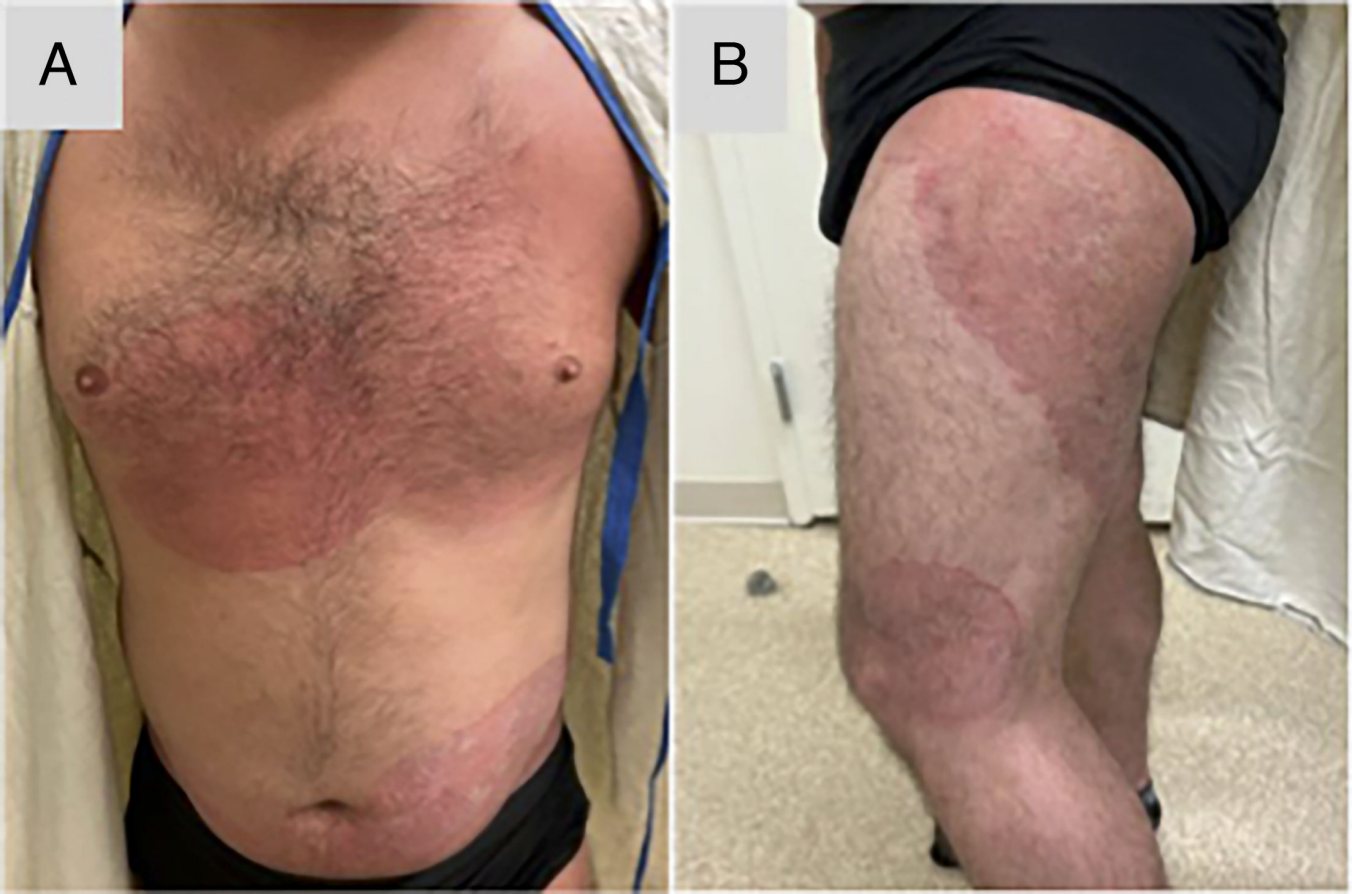
Annular, erythematous plaques with central clearing and overlying scale along the trunk (**A**) and left leg (**B**) of Patient A.

Examination revealed annular, erythematous, scaly plaques along the left arm, axillae, trunk, thighs, and buttocks (Fig. 1) and potassium hydroxide (KOH) scraping showing branched hyphae confirmed dermatophytosis. The extent and chronicity of the rash, along with the limited response to topical and oral antifungals, prompted suspicion of *T. indotineae*. Fungal culture yielded a *Trichophyton* species, which was identified as *T. indotineae* via ITS sequencing using an in-house validated assay, following CLSI MM18 guidelines (7). The isolate was also urease-negative, consistent with *T. indotineae*. Antifungal susceptibility testing (AFST) was performed at the Fungus Testing Laboratory at UT Health San Antonio and demonstrated a minimum inhibitory concentration (MIC) of 0.06 μg/mL for both terbinafine and itraconazole. He was initially prescribed an 8-week course of itraconazole 200 mg daily, which was extended for an additional month. He reported significant improvement at follow-up.

Three weeks later, his 24-year-old immunocompetent brother (Patient B) presented similarly. He had also immigrated from Honduras in 2020 and reported rash onset several months after arrival. Examination revealed large, scaly, erythematous plaques with annular morphology and coalescing papules distributed across the medial thighs, abdomen, back, and buttocks. KOH preparation again showed branched hyphae. A *Trichophyton* species was isolated in culture and identified as *T. indotineae* using the same ITS sequencing method. AFST yielded MICs of 0.015 μg/mL for terbinafine and 0.125 μg/mL for itraconazole. He began an 8-week course of oral itraconazole 200 mg daily and demonstrated clinical improvement at follow-up.

## DISCUSSION

Initially described as *Trichophyton mentagrophytes* ITS genotype VIII, *T. indotineae* was designated a separate species in 2020 based on ITS region sequencing of two terbinafine-resistant isolates from Indian and Nepalese patients (3). It has since emerged as a global dermatologic and public health concern due to its high rate of antifungal resistance, capacity for human-to-human transmission, and potential for misidentification in clinical laboratories (2, 3, 5–9). Speculation regarding its emergence has focused on widespread use of fixed-dose topical steroid-antifungal-antibacterial combinations and limited antifungal stewardship in endemic areas (2). Cases have now been reported in Europe and North America, including several states in the US (2–4).

We report two cases from Virginia, a state with no previously published cases, supporting its ongoing geographic spread. Both patients developed persistent pruritic rashes in 2020 after immigrating to the US, but *T. indotineae* was only suspected in 2024 due to worsening symptoms and antifungal treatment failure. Neither patient reported international travel post-immigration. This delay reflects diagnostic challenges and limited species-level identification capacity in most clinical labs. While pre-immigration acquisition is possible, prolonged symptoms and household exposure raise the possibility of US community transmission before broader recognition of *T. indotineae* in North America (2).

Clinically, *T indotineae* infections present as diffusely distributed, inflamed, pruritic plaques manifesting as tinea corporis, tinea cruris, or tinea faciei (2, 4, 6). Intense pruritus, prolonged infection, poor response to therapy, or relapse should raise suspicion for *T. indotineae*. Microscopy and culture cannot distinguish *T. indotineae* from *T. mentagrophytes* or *T. interdigitale* (3, 8). While urease negativity after 7–10 days can raise suspicion, ITS sequencing is required for a definitive diagnosis (8).

**Fig 2:**
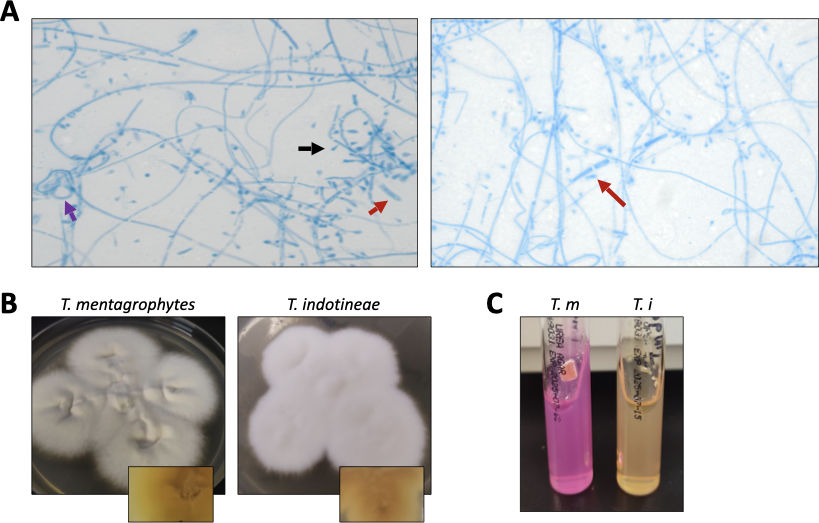
Morphological and culture characteristics of *Trichophyton indotineae*. (**A**) Lactophenol cotton blue tape preparation of *T. indotineae*. Purple arrow indicates spiral hyphae; red arrow, cigar-shaped to club-shaped macroconidia; black arrows, microconidia. (**B**) *T. indotineae* colonies on potato dextrose agar (PDA) after incubation at 30°C; inset shows reverse pigmentation. (**C**) Urease test on Christensen urea agar after 7 days at 30°C; urease-positive indicated by pink-red coloration.

Culture and microscopy features of *T. indotineae* and *T. mentagrophytes* are overlapping and nonspecific (8). Both organisms produce flat, white to beige, cottony to powdery colonies on Sabouraud dextrose agar and potato dextrose agar after 12–14 days of incubation at 30°C, often with yellow to brown reverse pigmentation (Fig. 2B). Microscopic evaluation from tape preparations stained with lactophenol cotton blue reveals small and large round microconidia in grape-like clusters, cigar- to club-shaped macroconidia with three to six septa, and occasional spiral hyphae in older cultures (Fig. 2A). These morphologic features do not reliably distinguish *T. indotineae* from other members of the *T. mentagrophytes/interdigitale* complex and should not be used for species-level identification.

In our laboratory, MALDI-TOF MS using the VITEK MS Prime (bioMerieux, KB v3.3.0) identified both isolates as part of the *T. mentagrophytes*/*interdigitale* complex with high confidence (99.6–99.9%), but lacked species-level resolution. Similarly, the MALDI Biotyper MBT HT Filamentous Fungi Module (Bruker Daltonics) does not currently include *T. indotineae* in its reference database, although it includes *T. mentagrophytes* group. This gap is consistent with published studies demonstrating poor sensitivity for *T. indotineae* in commercial libraries (11, 12). While curated spectral databases like MSI-2 and BCCM/IHEM offer enhanced performance, these are not routinely available in most clinical settings (11, 12).

In settings without molecular tools, adjunctive urease testing may help prioritize isolates. While *T. mentagrophytes* and *T. interdigitale* typically produce urease within 7–10 days, *T. indotineae* remains negative (Fig. 2C) (8). In this case, both isolates remained negative at 7 days, supporting the identification after suspicion had already been raised based on clinical history and morphologic findings. For laboratories without routine molecular testing, we propose a practical stepwise approach: (i) identify using MALDI-TOF MS, (ii) perform urease testing and read at day 7, and (iii) refer urease-negative isolates for sequencing and AFST if suspicion remains high.

Antifungal resistance is a hallmark of *T. indotineae*. Terbinafine resistance is commonly due to mutations in the squalene epoxidase gene which reduce drug binding (5, 6, 10). MICs of ≥0.5 µg/mL have been linked to clinical failure (5, 6). In our cases, susceptibility testing was performed using the CLSI M38 third edition broth microdilution method. Few US labs offer this service; the American Academy of Dermatology (AAD) lists only three: University Hospitals Cleveland, Wadsworth Center (mainly for NY providers), and UT Health San Antonio.

The isolate from Patient A showed the same MICs for terbinafine and itraconazole, while the isolate from Patient B had a lower MIC for terbinafine. Although these findings might suggest some retained terbinafine activity, their clinical relevance is limited by the lack of established breakpoints and the known disconnect between MIC values and treatment outcomes. In both cases, clinicians selected oral itraconazole based on the severity and chronicity of the infections. This choice aligns with guidance from the AAD, which supports initiating empirical therapy while awaiting confirmatory testing (5, 9). Oral itraconazole is the preferred treatment (5), with a generally accepted first-line treatment dose of 100–200 mg/day (7). Clinicians should be aware that an extended duration may be required, and patients often relapse (1, 2, 5).

Emerging reports suggest that azole resistance may complicate the management of *T. indotineae* infections. Ebert et al. reported that approximately one-quarter of isolates in a large Indian cohort were resistant to itraconazole and/or voriconazole (13). More recently, Yamada et al. identified CYP51B gene amplification as a mechanism of azole resistance, showing that overexpression alone can confer resistance (14).

Finally, patients should also be counseled regarding the highly transmissible nature of *T indotineae* infections through physical contact, as well as the potential for indirect transmission via surfaces in shared living environments, bedding, and body linen (5).

### Conclusion

While the prevalence of *T. indotineae* remains unclear, these cases in brothers residing in Virginia who denied international travel support its continued geographic expansion and raise concern for possible local transmission, though acquisition in Central America prior to their arrival in the United States remains a possibility. The geographic spread, diagnostic delays, and inappropriate use of topical steroids depicted here underscore the importance of increased clinical awareness. Recognition remains limited in many clinical microbiology laboratories due to poor species-level resolution by routine methods and inconsistent access to molecular or susceptibility testing. *T. indotineae* represents an emerging dermatologic and public health threat whose resistance profile, diagnostic complexity, and potential for community transmission necessitate coordinated laboratory workflows, broader access to molecular diagnostics, and continued surveillance.

## Data Availability

Sequence data for *T. indotineae* isolates were deposited in GenBank with accession numbers PX854879 and PX854880.

## References

[1] Spivack S, Gold JAW, Lockhart SR, Anand P, Quilter LAS, Smith DJ, Bowen B, Gould JM, Eltokhy A, Gamal A, Retuerto M, McCormick TS, Ghannoum MA. 2024. Potential sexual transmission of antifungal-resistant Trichophyton indotineae. Emerg Infect Dis 30:807–809. doi:10.3201/eid3004.240115 38437706 PMC10977831

[2] Caplan AS. 2021. Notes from the field: first reported US cases of tinea caused by Trichophyton indotineae—New York City, December 2021–March 2023. MMWR Morb Mortal Wkly Rep 72:536–537. doi:10.15585/mmwr.mm7219a4 PMC10208369 37167192

[3] Chowdhary A, Singh A, Kaur A, Khurana A. 2022. The emergence and worldwide spread of the species Trichophyton indotineae causing difficult-to-treat dermatophytosis: a new challenge in the management of dermatophytosis. PLoS Pathog 18:e1010795. doi:10.1371/journal.ppat.1010795 36173977 PMC9521800

[4] Cañete-Gibas CF, Mele J, Patterson HP, Sanders CJ, Ferrer D, Garcia V, Fan H, David M, Wiederhold NP. 2023. Terbinafine-resistant dermatophytes and the presence of Trichophyton indotineae in North America. J Clin Microbiol 61:e0056223. doi:10.1128/jcm.00562-23 37432126 PMC10446870

[5] Uhrlaß S, Verma SB, Gräser Y, Rezaei-Matehkolaei A, Hatami M, Schaller M, Nenoff P. 2022. Trichophyton indotineae-an emerging pathogen causing recalcitrant dermatophytoses in india and worldwide-a multidimensional perspective. J Fungi (Basel) 8:757. doi:10.3390/jof8070757 35887512 PMC9323571

[6] Caplan AS, Todd GC, Zhu Y, Sikora M, Akoh CC, Jakus J, Lipner SR, Graber KB, Acker KP, Morales AE, Rolón RMM, Westblade LF, Fonseca M, Cline A, Gold JAW, Lockhart SR, Smith DJ, Chiller T, Greendyke WG, Manjari SR, Banavali NK, Chaturvedi S. 2024. Clinical course, antifungal susceptibility, and genomic sequencing of Trichophyton indotineae. JAMA Dermatol 160:701. doi:10.1001/jamadermatol.2024.1126 38748419 PMC11097098

[7] Clinical and Laboratory Standards Institute (CLSI). 2018. MM18—interpretive criteria for identification of bacteria and fungi by targeted DNA sequencing; approved guideline. 2nd ed. CLSI.

[8] Marbaniang YV, Leto D, Almohri H, Hasan MR. 2025. Treatment and diagnostic challenges associated with the novel and rapidly emerging antifungal-resistant dermatophyte, Trichophyton indotineae J Clin Microbiol 63:e0140724. doi:10.1128/jcm.01407-24 40497720 PMC12153305

[9] American Academy of Dermatology Association. 2025. Preventing and treating Trichophyton indotineae. American Academy of Dermatology Association. Available from: https://www.aad.org/member/clinical-quality/clinical-care/emerging-diseases/dermatophytes/preventing-treating-trichophyton-indotineae

[10] Sacheli R, Hayette MP. 2021. Antifungal resistance in dermatophytes: genetic considerations, clinical presentations and alternative therapies. J Fungi (Basel) 7:983. doi:10.3390/jof7110983 34829270 PMC8622014

[11] De Paepe R, Normand A-C, Uhrlaß S, Nenoff P, Piarroux R, Packeu A. 2024. Resistance profile, terbinafine resistance screening and MALDI-TOF MS identification of the emerging pathogen Trichophyton indotineae. Mycopathologia 189:29. doi:10.1007/s11046-024-00835-4 38483637 PMC10940462

[12] Normand AC, Moreno-Sabater A, Jabet A, Hamane S, Cremer G, Foulet F, Blaize M, Dellière S, Bonnal C, Imbert S, Brun S, Packeu A, Bretagne S, Piarroux R. 2022. MALDI-TOF mass spectrometry online identification of Trichophyton indotineae using the MSI-2 application. J Fungi (Basel) 8:1103. doi:10.3390/jof8101103 36294668 PMC9604624

[13] Ebert A, Monod M, Salamin K, Burmester A, Uhrlaß S, Wiegand C, Hipler U-C, Krüger C, Koch D, Wittig F, Verma SB, Singal A, Gupta S, Vasani R, Saraswat A, Madhu R, Panda S, Das A, Kura MM, Kumar A, Poojary S, Schirm S, Gräser Y, Paasch U, Nenoff P. 2020. Alarming India-wide phenomenon of antifungal resistance in dermatophytes: a multicentre study. Mycoses 63:717–728. doi:10.1111/myc.13091 32301159

[14] Yamada T, Yaguchi T, Maeda M, Alshahni MM, Salamin K, Guenova E, Feuermann M, Monod M. 2022. Gene amplification of CYP51B: a new mechanism of resistance to Azole compounds in Trichophyton indotineae. Antimicrob Agents Chemother 66:e0005922. doi:10.1128/aac.00059-22 35546111 PMC9211412

